# The Lung Macrophage in SARS-CoV-2 Infection: A Friend or a Foe?

**DOI:** 10.3389/fimmu.2020.01312

**Published:** 2020-06-05

**Authors:** Zaid Abassi, Yara Knaney, Tony Karram, Samuel N. Heyman

**Affiliations:** ^1^Department of Physiology and Biophysics, Rappaport Faculty of Medicine, Technion-Israel Institute of Technology, Haifa, Israel; ^2^Laboratory Medicine, Rambam Health Care Campus, Haifa, Israel; ^3^Department of Vascular Surgery, Rambam Health Care Campus, Haifa, Israel; ^4^Department of Medicine, Hadassah Hebrew University Hospital, Jerusalem, Israel

**Keywords:** SARS-CoV-2, macrophages, ACE2, lung, acute respiratory distress syndrome, defense, reservoir

## Abstract

Respiratory, circulatory, and renal failure are among the gravest features of COVID-19 and are associated with a very high mortality rate. A common denominator of all affected organs is the expression of angiotensin-converting enzyme 2 (ACE2), a protease responsible for the conversion of Angiotensin 1-8 (Ang II) to Angiotensin 1-7 (Ang 1-7). Ang 1-7 acts on these tissues and in other target organs via Mas receptor (MasR), where it exerts beneficial effects, including vasodilation and suppression of inflammation and fibrosis, along an attenuation of cardiac and vascular remodeling. Unfortunately, ACE2 also serves as the binding receptor of SARS viral spike glycoprotein, enabling its attachment to host cells, with subsequent viral internalization and replication. Although numerous reports have linked the devastating organ injuries to viral homing and attachment to organ-specific cells widely expressing ACE2, little attention has been given to ACE-2 expressed by the immune system. Herein we outline potential adverse effects of SARS-CoV2 on macrophages and dendritic cells, key cells of the immune system expressing ACE2. Specifically, we propose a new hypothesis that, while macrophages play an important role in antiviral defense mechanisms, in the case of SARS-CoV, they may also serve as a Trojan horse, enabling viral anchoring specifically within the pulmonary parenchyma. It is tempting to assume that diverse expression of ACE2 in macrophages among individuals might govern the severity of SARS-CoV-2 infection. Moreover, reallocation of viral-containing macrophages migrating out of the lung to other tissues is theoretically plausible in the context of viral spread with the involvement of other organs.

The reported clinical manifestations of Covid-19 keep growing steadily. Respiratory, circulatory, and renal failure are among its gravest features, and the mortality rate is very high (1–3). Other organ involvement includes the gastrointestinal tract (manifested as diarrhea and vomiting) (4, 5), gonads [impaired male fertility (6)], and nervous system (7). A common denominator of all affected organs is the expression of angiotensin-converting enzyme 2 (ACE2) (8, 9). ACE2 is a transmembranal protease responsible for the conversion of Angiotensin 1-8 (Ang II) to Angiotensin 1-7 (Ang 1-7) (10). The latter acts on these tissues and in other target organs via Mas receptor (MasR), where it exerts beneficial effects, including vasodilation and suppression of inflammation and fibrosis (8, 9). Ang 1-7 also induces diuresis/natriuresis, preserves renal function, and attenuates cardiac and vascular remodeling (11).

Unfortunately, ACE2 also serves as the binding receptor of SARS viral spike glycoprotein, enabling its attachment to host cells, with subsequent viral internalization and replication (12–14). So far, numerous reports have linked the devastating organ injuries to viral homing and attachment to organ-specific cells widely expressing ACE2; however, little attention has been given to the immune system. The following short commentary outlines potential adverse effects of SARS-CoV2 on macrophages and dendritic cells, key cells of the immune system, which also express ACE2 (15, 16).

Macrophages and dendritic cells are ubiquitous in human organs with a substantial abundance in the lungs. There are two distinct populations of pulmonary macrophages: alveolar macrophages, which reside in proximity to type I and type II epithelial alveolar cells, and interstitial macrophages, which are preferentially abundant between the microvascular endothelium and alveolar epithelium zone (17) (Figure 1). Various pathogens and noxious materials reaching the lungs provoke an innate immune response of the pulmonary parenchyma that is characterized by the differentiation of bone-marrow-derived monocytes into alveolar macrophages, which serve as a first-line defense against invading organisms. Both alveolar and interstitial macrophages can be divided into two functional phenotypes. The first is made up of classically activated macrophages (M1 macrophage), which are activated by pathogen-associated molecular patterns (PAMPs) that are also expressed by viruses. Their activity is then promoted by Th1 cells. The second population includes the alternatively activated macrophages (M2 macrophage), which are activated by Th2 cells by means of IL-4 and IL-13 (17). M1 macrophages induce recruitment of immune cells into the lung parenchyma. In contrast, activation of M2 macrophages triggers the release of anti-inflammatory cytokines, which restrict inflammation and promote tissue repair (17). Dendritic cells play a keen role in the inflammatory process as evident by their responsibility for presentation of antigens, regulation of T-cell reactions to antigen, and the intensity of the inflammatory response. Activation of dendritic cells induces their expression of co-stimulation molecules such as CD80. Viral infections provoke monocytal-enhanced proinflammatory signaling molecules and antiviral responses, as have been shown with influenza, herpes, and Zika viruses (18). It has recently been suggested that enhanced activity of pro-inflammatory macrophages in part of the COVID-19 patients leads to accelerated production of inflammatory cytokines and chemokines, and among them is CXCL10, which leads to cytokine storms. This has mostly been observed in subjects with poor prognosis (19, 20). In general, short living monocytes/macrophages are able to remarkably limit viral replication. However, that does not preclude these cells from serving as a permissive system and/or as a viral reservoir (18). Support for this notion is derived from the fact that these cells serve as the first line of defense upon encountering viral infection. However, viral infection may convert these cells into long living macrophages (Mϕ) and promote their migration into tissues where they become infected resident cells. Finally, since SARS viruses, including SARS-CoV2, utilize ACE2 as a tight binding site with high affinity (12–14), pulmonary macrophages that express ACE2 may permeate pulmonary invasion during SARS infection. Indeed, we have previously shown that monocytes/macrophages express ACE2 (15). Furthermore, monocyte-derived macrophages from patients with CHF exhibit profoundly increased ACE2 expression after treatment with spironolactone, a mineralocorticoid blocker. The beneficial impact of upregulated ACE2 in CHF patients is evident by attenuated oxidative stress, as expressed by reduced lipid peroxide content, superoxide ion release, and low-density lipoprotein oxidation. Similarly, mice treated with eplerenone, another mineralocorticoid blocker, displayed enhanced cardiac ACE2 activity in parallel to increased ACE2 activity in macrophages (15). Interestingly, macrophages also express furin and TMPRSS2, two enzymes involved in the exposure of the binding and effusion sites of the SARS virus (21, 22), as well as ADAM 17, which acts as sheddase of ACE2 (23). In the presence of all components of viral binding and activation, the virus can theoretically replicate in human macrophages and dendritic cells, triggering the aberrant production of proinflammatory cytokines/chemokines, as is the case with MERS-CoV (24). In contrast, some studies ruled out SARS-CoV viral replication in human macrophages (25). Despite abortive infection, characterized by infection without replication, SARS-CoV infection of human macrophages induced the expression of proinflammatory chemokines, whereas antiviral cytokine production was largely absent (26, 27). Studies also demonstrated that human dendritic cells are susceptible to SARS-CoV but unable to support viral replication (28).

**Figure 1:**
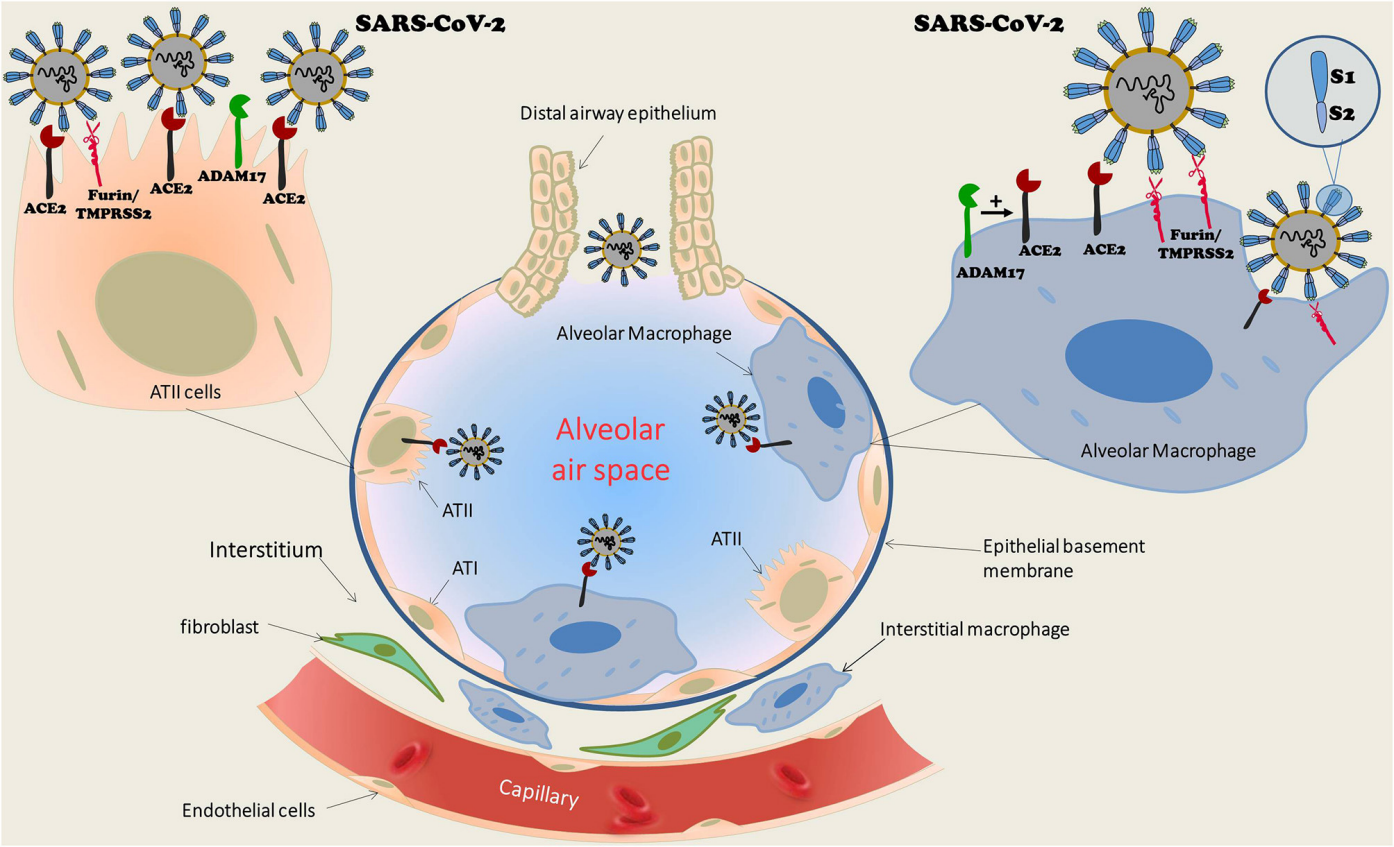
Schematic structure of pulmonary alveoli with diverse cell types, including cuboid ciliated epithelial cells along bronchioles, alveolar type I (ATI) and type II epithelial cells (ATII), and macrophages. The latter are ubiquitous in the lungs and consist of two distinct populations: alveolar macrophages, which reside in proximity to ATI and ATII, as well as interstitial macrophages, which are abundant between the microvascular endothelium and alveolar epithelium zone. Alveolar macrophages as well as ATII express ACE2, the binding receptor of SARS-CoV-2. In addition, both cell types express TMPRSS2/Furin, which are also required for viral attachment. It exposes the viral receptor binding protein (RBP) localized to S-glycoprotein (S1 domain of the viral spike) and reveals the effusion site on the S2 domain. Although SARS-CoV-2 replication in ATII cells is well-documented, a similar process was not confirmed in alveolar macrophages. While some studies suggested such a replication along triggering aberrant production of proinflammatory cytokines/chemokines, as is the case with MERS-CoV, others reports ruled out SARS-CoV viral replication in human macrophages. ACE2, Angiotensin converting enzyme 2; ATI, Alveolar epithelial cells type I; ATII, Alveolar epithelial cells type II; TMPRSS2, Transmembrane protease, serine 2.

COVID-19 morbidity and mortality are markedly increased in specified populations, namely aged and diabetic individuals, patients with chronic obstructive lung disease (COPD) or congestive heart failure (CHF) (3), and perhaps among patients on inhibitors of the renin angiotensin aldosterone system (RAAS) (3, 29). These observations might be linked with increased numbers of alveolar macrophages (AM) in such patients or with alterations in the AM phenotype. Indeed, increased numbers of AM in bronchoalveolar lavage (BAL) were detected in humans with COPD in proportion to their disease severity (30). Increased numbers of AM in BAL were also noted in mice following protracted exposure to diesel exhaust particles (31), and this is a consistent finding related to air pollution (32). Increased numbers of AM in BAL were noted also in aged vs. young rodents, and this difference was particularly prominent following exercise (31, 33). Furthermore, aging was associated with an altered phenotypic distribution of AM and with reduced bactericidal capacity in mice (34). AM were also more abundant in mice subjected to heart failure following augmented hypertension (35) or in models of dilated cardiomyopathy, combined with exercise (36). It was also noted in diabetic mice—associated with intensified indices of oxidative stress—yet these abnormalities were prevented by long-term treatment with angiotensin 1-7 (37). Furthermore, as with aging, experimental diabetes is associated with altered phenotype expression of AM (38) with decreased bactericidal capabilities (39). Taken together, increased susceptibility to serious COVID-19 infection occurs in clinical scenarios associated with increased AM population. It is tempting to suggest that conditions characterized by increased numbers of alveolar macrophages in the lower respiratory tract might facilitate homing of COVID-19 by their abundant expression of ACE2.

Collectively, in light of these observations, we propose a new hypothesis that while macrophages play an important role in antiviral defense mechanisms, in the case of SARS-CoV. they may also serve as a Trojan horse, enabling viral anchoring specifically within the pulmonary parenchyma. In other words, the unique expression of ACE2 in macrophages may, paradoxically, enable pulmonary invasion by SARS-CoV, facilitating engraftment, and inducing protracted local and systemic uncontrolled inflammatory responses (40). It is tempting to assume that diverse expression among individuals of ACE2 in macrophages might govern the severity of SARS-CoV-2 infection. Moreover, besides direct invasion caused by viremia, reallocation of viral-containing macrophages migrating out of the lung to other tissues is theoretically plausible in the context of viral spread with the involvement of other organs. To some extent, this setup resembles a comparable phenomenon, termed “the macrophage paradox,” were intracellular bacterial pathogens preferentially replicate within macrophages (41). Our hypothesis is further supported by a recent report of post-mortem findings in patients succumbing to SARS-CoV, showing ACE2 expression and viral nucleocaspid protein in CD169+ macrophages in lymph nodes and in the spleen (42). The attenuation of experimental lethal SARS in rodents by monocyte/macrophage depletion (43) is also to some extent in line with our hypothesis. On the other hand, a recent study demonstrated that proinflammatory monocyte-derived macrophages were abundant in bronchoalveolar lavage obtained from patients with severe SARS-CoV-2 pulmonary involvement, as compared with those with moderate disease (44). In fact, it has been suggested that monocyte-derived macrophages replace damaged infected alveolar macrophages in severe cases, and likely do not indicate the substitution of alveolar cells migrating to other tissues (44). This possibility is supported by documented death of infected macrophages *in vitro*. Furthermore, increasing evidence suggests that aberrant myeloid responses may underlie some of the COVID-19 hallmark manifestations, including acute respiratory distress syndrome (ARDS), cytokine release syndrome, and lymphopenia (45). In this context, recent studies in humanized hACE2 mice demonstrated that these animals exhibited characteristic alveolar interstitial pneumonia, with infiltration of lymphocytes and monocytes and accumulation of macrophages in the alveolar lumen (46), corresponding with the clinical findings (47). Moreover, primate and clinical data on SARS-CoV-1 have also shown that virus spike-specific IgG responses exacerbate ARDS due to repolarization of alveolar macrophages into pro-inflammatory phenotypes and enhanced recruitment of inflammatory monocytes via CCL2 and IL-8 (48). Collectively, it is obvious that the immune system undergoes profound and complex alterations during symptomatic COVID-19 disease, including migration of inflammatory monocytes with CD14+IL-1β+ monocytic expansion, as elegantly summarized by Vabret et al. (48) in a comprehensive review on the fast evolving field of COVID-19 immunology.

Finally, it should be emphasized that our hypothesis is not sufficiently evidence based. We still lack carefully produced data about the susceptibility of tissue macrophages to SARS-CoV-2 and their capacity to produce *de novo* infectious viral particles. Additional studies are also required to assess reduced ACE2 expression following macrophage invasion by SARS-CoV-2 and the plausible causative association that links modified macrophages to the evolving inflammatory storm.

## Data Availability Statement

The original contributions presented in the study are included in the article/supplementary material, further inquiries can be directed to the corresponding author/s.

## Author Contributions

ZA, YK, TK, and SH equally participated in the design, execution of the view of point, drafted the manuscript, participated in critical discussions and revised the manuscript. YK and ZA prepared the figure. ZA supervised the project.

## Conflict of Interest

The authors declare that the research was conducted in the absence of any commercial or financial relationships that could be construed as a potential conflict of interest.
